# Supplementary figures and images for: Sense-overlapping lncRNA as a decoy of translational repressor protein for dimorphic gene expression
Source: PLoS Genet. 2021 Jul 28;17(7):e1009683. doi: 10.1371/journal.pgen.1009683 (PMC8351930; doi:10.1371/journal.pgen.1009683)

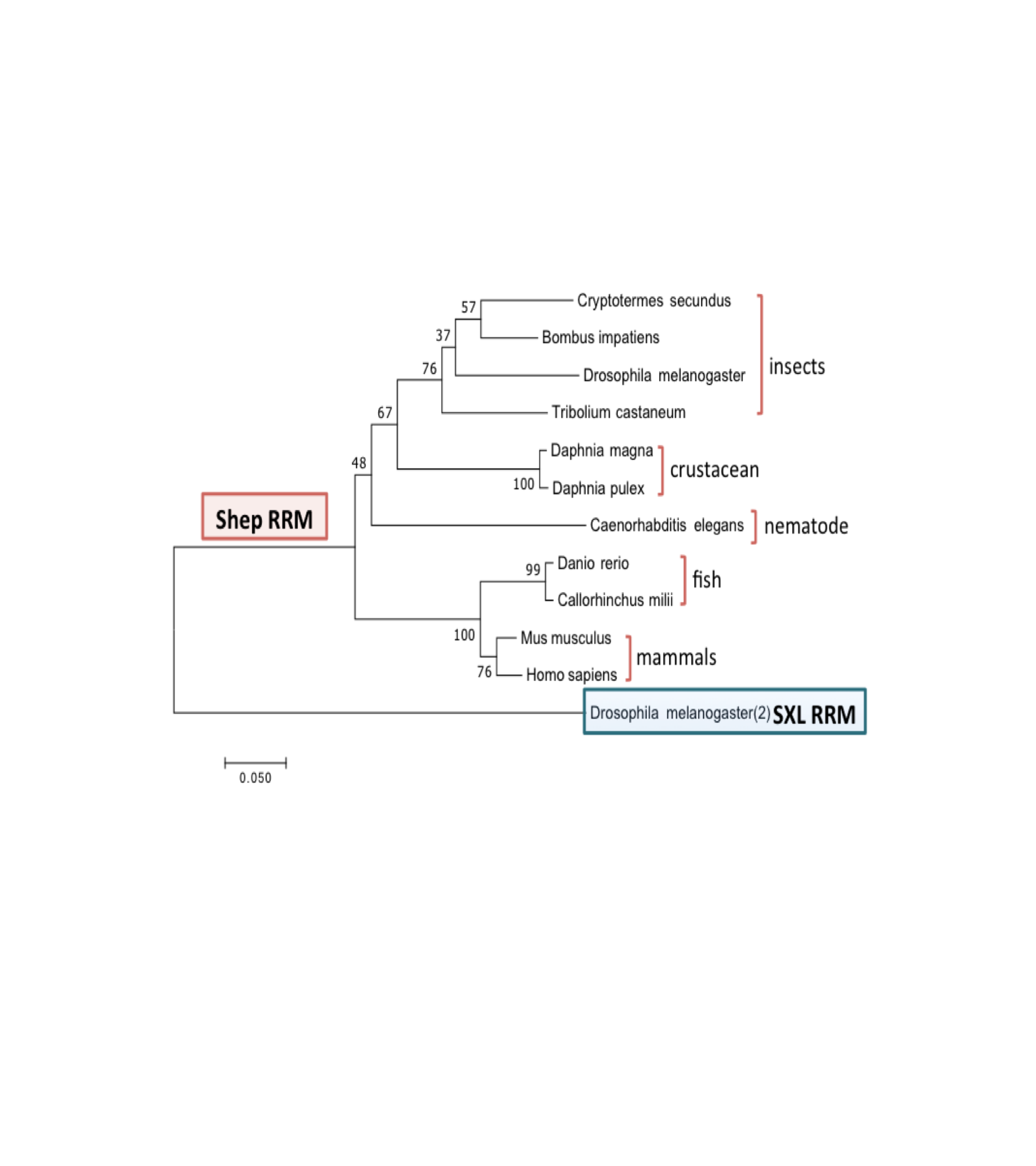

Supplement: S1 Fig — RRMs of Shep orthologs are labeled with red while the Sex-lethal (SXL) RRM is boxed in blue. The percentages of the replicate tree in which the associated taxa clustered together in the bootstrap test (500 replicates) are shown next to the branches. The bar indicates branch length and corresponds to the mean number of the differences (P<0.05) per residue along each branch. Evolutionary distances were computed using the p-distance method. (TIF) [file pgen.1009683.s001.tif]

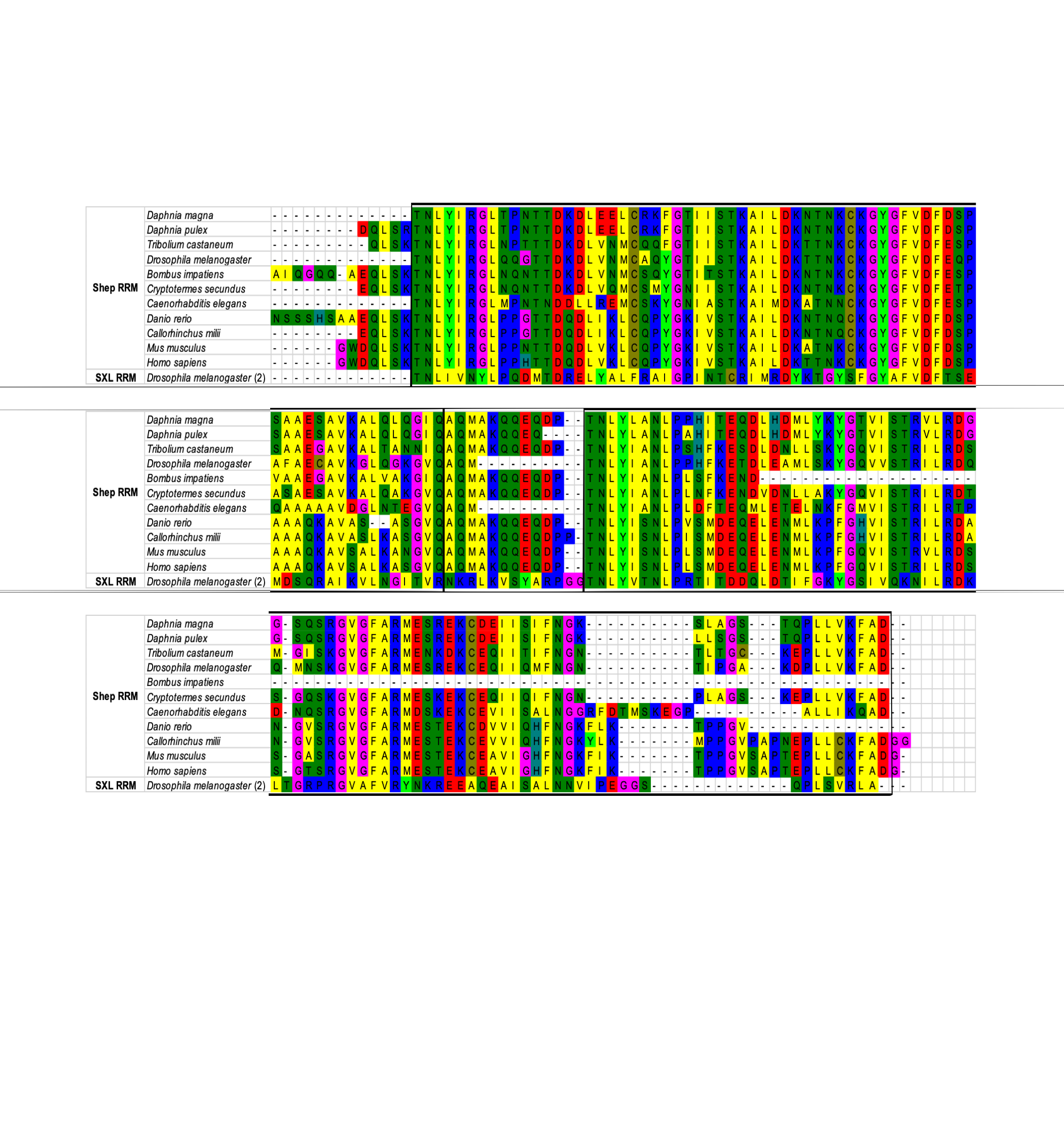

Supplement: S2 Fig — Alignment of the RNA Recognition Motifs (RRMs) of the different Shep orthologs from different organisms. The color is based on the physicochemical property of the amino acid-based on ClustalW. The boxes represent the position of the two RRM regions. (TIF) [file pgen.1009683.s002.tif]

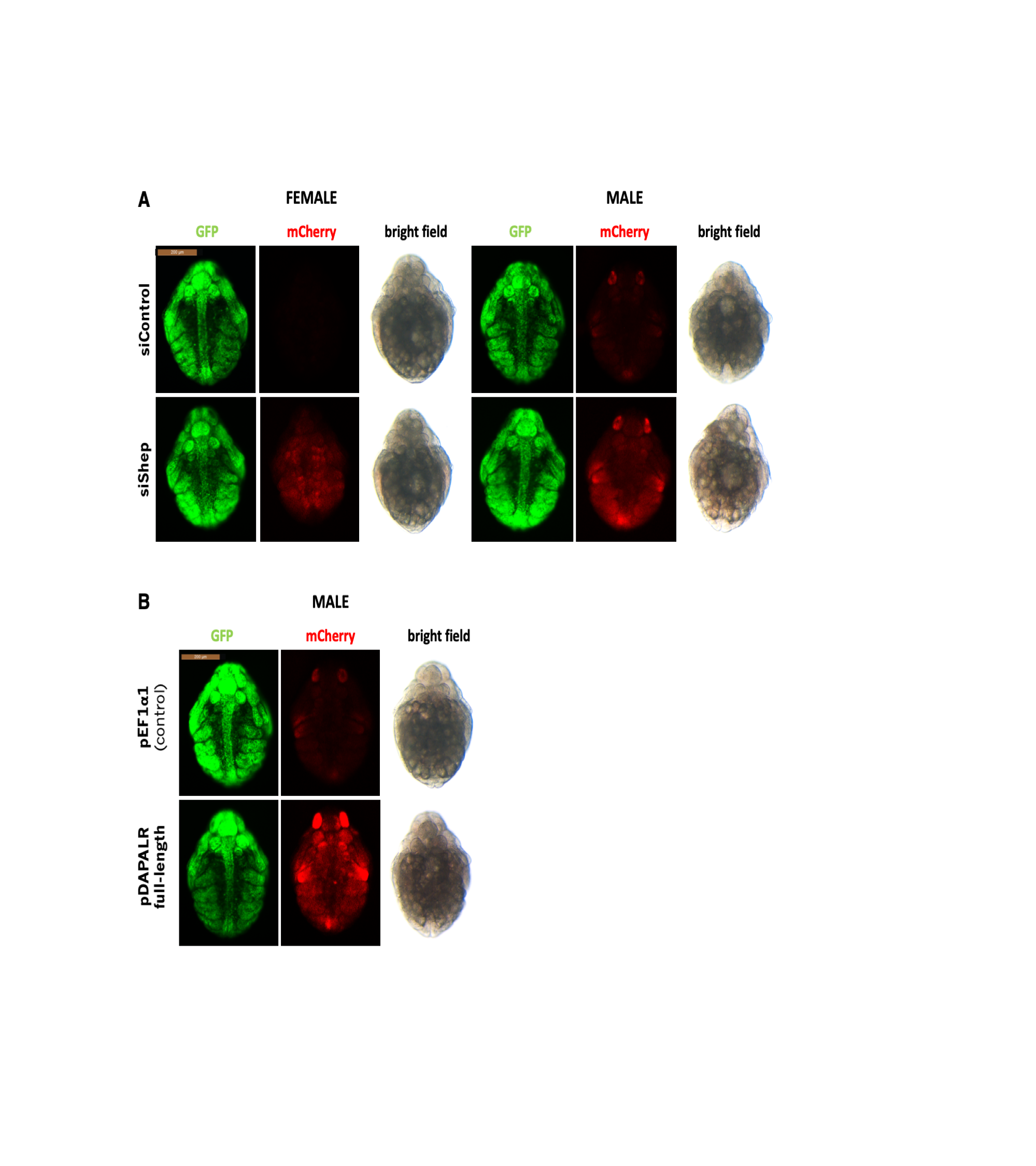

Supplement: S3 Fig — (A) Ventral view of female and male embryos of Dsx1 reporter strain injected with control siRNA and Shep siRNA and observed at 30 h after injection. mCherry fluorescence allowed visualization of Dsx1 expression while GFP fluorescence in the nucleus enabled observation of body structures. The bright field images were used to understand the localization pattern of mCherry expression. (B) Ventral view of male embryos of Dsx1 reporter strain injected with control plasmid and DPALR-expressing plasmid observed at 30 h after injection. (TIF) [file pgen.1009683.s003.tif]

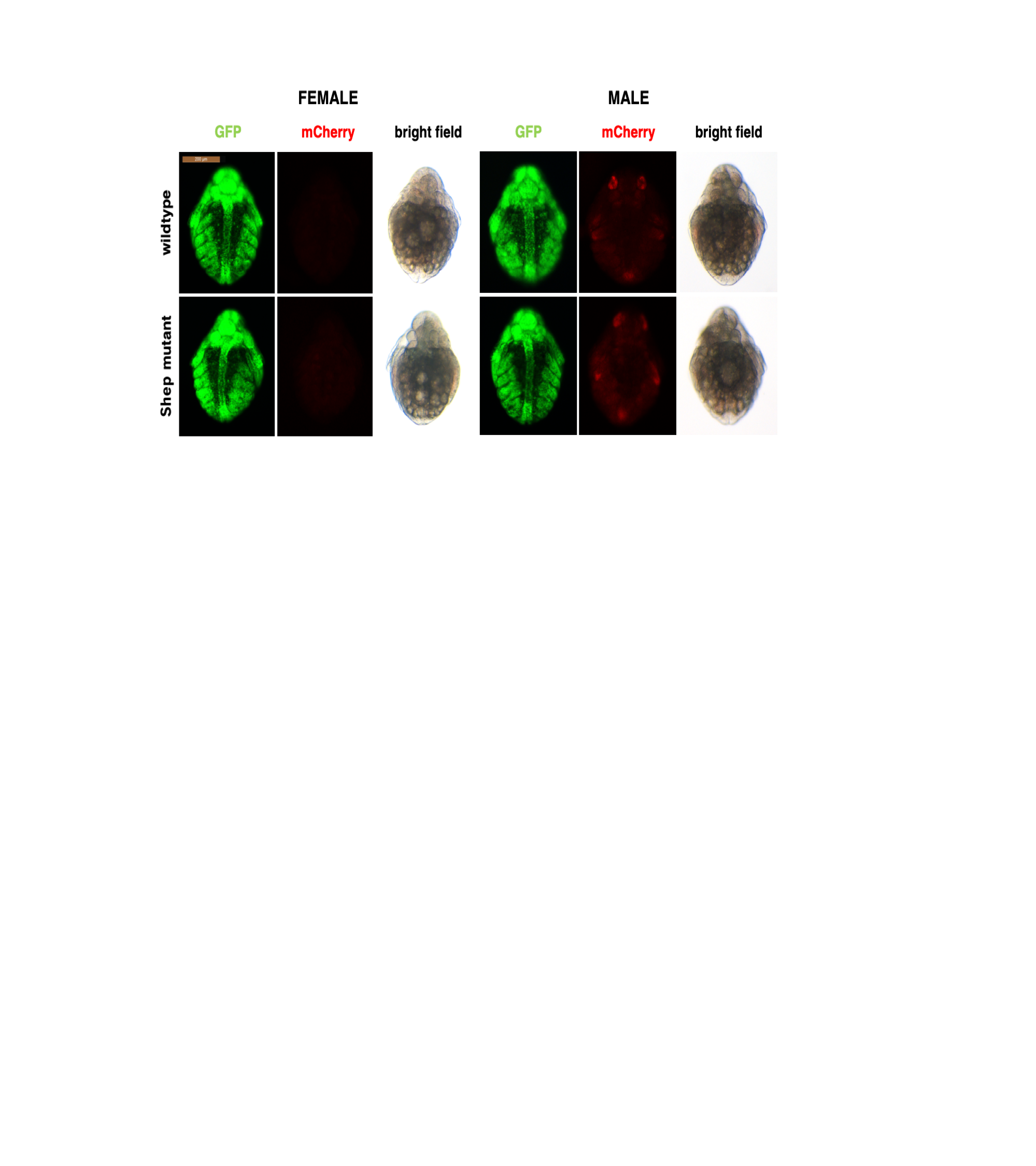

Supplement: S4 Fig — Ventral view of female and male embryos of Shep mutant line observed at 30 h after ovulation. mCherry fluorescence allowed visualization of Dsx1 expression while GFP fluorescence in the nucleus enabled observation of body structures. The bright field images were used to understand the localization pattern of mCherry expression. (TIF) [file pgen.1009683.s004.tif]

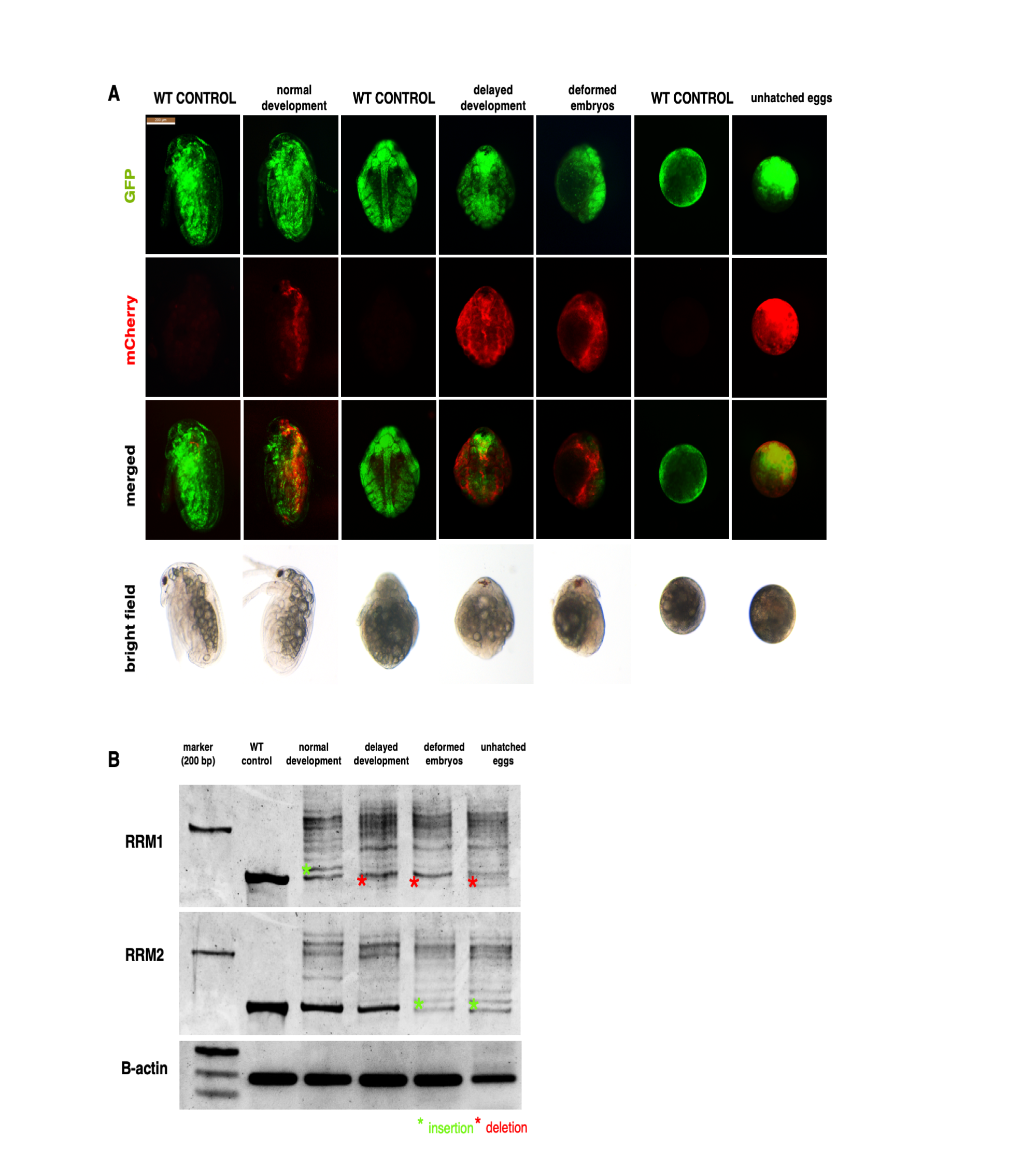

Supplement: S5 Fig — (A) Ventral and lateral views of the different phenotypes observed after injection of Cas9 and Shep-targeting gRNAs: (from L to R) normal development, delayed development, abnormal development and unhatched egg. Phenotypes of uninjected embryos showing normal development were also shown as control phenotypes at each stage. mCherry fluorescence allowed visualization of Dsx1 expression while GFP fluorescence in the nucleus enabled observation of body structures. The merged images of mCherry and GFP were used to understand the localization pattern of mCherry expression. Bright field showed photos of embryos taken using visible light. Scale bar = 200 μm. (B) PAGE analysis of PCR products by genomic PCR to amplify the region targeted by each RRM-targeting gRNAs. Asterisks show the genomic mutations in RRM1- and RRM2-coding sequences of embryos showing the different phenotypes after Shep mutagenesis. (TIF) [file pgen.1009683.s005.tif]

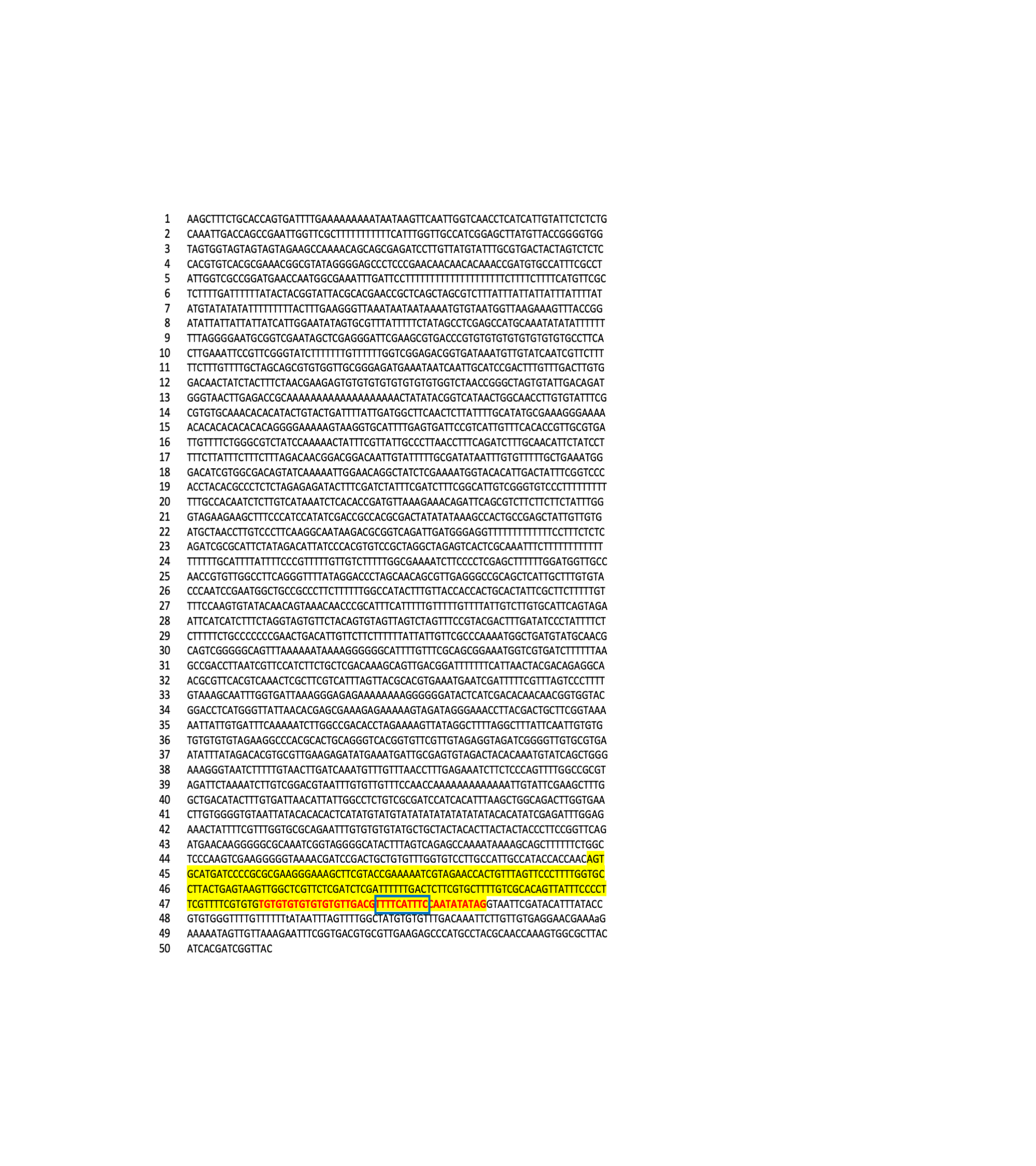

Supplement: S6 Fig — The full sequence of DAPALR is shown. Its overlapping region with Dsx1 5´ UTR (205 bp) is highlighted in yellow. Colored in red is the 40 nt core element of DAPALR harboring the Shep binding site. The blue box indicates the 10 bp of the sequece subjected to deletion of the Shep binding site for the in vitro and in vivo experiments. (TIF) [file pgen.1009683.s006.tif]
