# Supplementary material for: Sense-overlapping lncRNA as a decoy of translational repressor protein for dimorphic gene expression
Source: PLoS Genet. 2021 Jul 28;17(7):e1009683. doi: 10.1371/journal.pgen.1009683 (PMC8351930; doi:10.1371/journal.pgen.1009683)
Supplement: S2 Table — (DOCX) [file pgen.1009683.s008.docx]

**S2 Table. Summary of mutagenesis experiment**
