# Supplementary material for: Sense-overlapping lncRNA as a decoy of translational repressor protein for dimorphic gene expression
Source: PLoS Genet. 2021 Jul 28;17(7):e1009683. doi: 10.1371/journal.pgen.1009683 (PMC8351930; doi:10.1371/journal.pgen.1009683)
Supplement: S3 Table — (DOCX) [file pgen.1009683.s009.docx]

| **target gene** | **Forward primer (5′-3′)** | **Reverse primer (5′-3′)** |
| --- | --- | --- |
| ***Shep*** | AACCTCTACATTCGCGGACT | GTAGCCTTTGCACTTGTTGG |
| ***Dsx1*** | AAGTTTGGTGTAGGGGAGGATGAG | CCATTCATCATTACCAAATCCCTTC |
| ***L32*** | GACCAAAGGGTATTGACAACAGA | CCAACTTTTGGCATAAGGTACTG |
| ***L8*** | GGTACTATTGTTTGCAATGTTGAGG | GTCTTCTTGGTATCGGTATTGTGAC |
| ***Cyclophilin*** | GACTTTCCACCAGTGCCATT | AACTTTCCATCGCATCATCC |
| **Bait RNA for FLAG pulldown** | GCTTTTGTCGCACAGTTATTTC | CTTCCAGCGGATAGAATGG |
| ***tRNA-Met*** | GAGCTTGTATAGTTTAATTGGTTAA | TACTTGTAGAAGGAATTGAACCTTA |
| ***tRNA-Phe*** | GGACTTAGCTCAGTTGGGA | GAACTCTGTGGATCGAACA |

**S3 Table. Primer sequences for RT-qPCR**.
