# Supplementary material for: Sense-overlapping lncRNA as a decoy of translational repressor protein for dimorphic gene expression
Source: PLoS Genet. 2021 Jul 28;17(7):e1009683. doi: 10.1371/journal.pgen.1009683 (PMC8351930; doi:10.1371/journal.pgen.1009683)
Supplement: Attachment — Submitted filename: renamed_43918.docx [file pgen.1009683.s010.docx]

**Editor's comment: The primary issue seem to be the lack of strong biochemical data to infer that shep interacts directly with Dsx or DAPALR. In the revised version, additional experimental evidence that support translational repression is encouraged.**

**Response**:

Thank you for the comment. We conducted the *in vitro* translation and luciferase assay to show that Shep only represses *Dsx1* translation in the presence of the TGE-like element. Deletion of the TGE element resulted in no change in the translation efficiency. We also conducted the FLAG pulldown experiment to show that the Shep directly binds to the TGE-like element *in vitro*. In this experiment, we proved that Shep can pull down the RNA with the TGE element and this interaction was inhibited in the absence of the TGE element. These results were shown in Fig 6A and 6B and discussed in Lines 264-307.

Responses to Reviewers

**Reviewer #1:** The manuscript by Perez et al presents a potentially interesting mode of translational regulation via a long non-coding RNA (lncRNA). The authors present data that suggest that the protein shep inhibits Dsx1 alpha isoform translation by the binding to a specific RNA element in the 5’ UTR called the TGE. Interestingly, there is a sense lncRNA called DAPALR, produced from the same region and containing a TGE, which is proposed to sequester shep and would therefore regulate Dsx1 translation. While this represents an interesting role for lncRNA regulation of gene expression, unfortunately the current manuscript is not well presented, the methods are inadequately described and direct evidence of shep binding to the DAPALR and Dsx1 RNA is lacking.

Response:

Thank you for reviewing this manuscript. We really appreciate all your suggestions and herewith is our humble and careful response to your comments below.

Major comments

**1) It would be very useful if Figure 1 had a simple diagram displaying the orientation of the DAPALR lncRNA with the Dsx1 alpha mRNA and an overall layout of the Dsx1 genomic region including the different isoforms.**

Response:

Thank you for the suggestion. We added the diagram showing the genomic organization of *Dsx1* including the position and orientation of the two isoforms and the lncRNA *DAPALR* in Fig 1A.

**2) Lines 51-53 – The statement “DAPALR is transcribed from upstream of the transcription start site and overlaps with the 5´ UTR of the Dsx1 alpha isoform, both of which are exclusively expressed in males (Kato et al, 2018, 2011).” needs clarification. Is only the Dsx1 alpha isoform expressed in males and other Dsx1 isoforms exist and are expressed in both males and females? How many Dsx1 isoforms are there and what is their expression in males and females. Does DAPALR only overlap the 5’ UTR of the Dsx1 alpha isoform and not the other isoforms?**

Response (Fig 1A, Lines 61-66):

We apologize for the confusing statement. The two isoforms of *Dsx1, α* and ß, are both exclusively expressed in males (Kato *et al*, 2011). *DAPALR*, like *Dsx1*, is also only expressed in males but it only overlaps with the 5’ UTR of the *Dsx1α* isoform (Kato *et al*, 2018). This can be seen in Fig 1A and we have also revised the mentioned statement in Lines 61-66.

**3) The description of the RNA pulldown and mass spectrometry methods lack detail (Lines 249-258). The Adachi & Natsume, 2015 reference the authors cite is not open access so it would be difficult for people to see the methods this work is based on. In any case, even with access to this reference key specific details used here are missing. What is the exact 205bp sequence used to make the RNA? How were cell lysates made from female larvae? What were the buffers used for the pulldown, washing and elution? Finally, how was the mass spectrometry carried out? For example, how were pulldown samples prepared for mass spectrometry, what type of mass spectrometry was used, how was the mass spectrometry data analysed and what were the criteria for identifying the three candidate proteins with a higher probability for binding to the overlapping sequence of DAPALR? Also the full mass spectrometry data should be presented as a supplementary file or a link to the data should be given.**

Response (Lines 78-84, 396-426, S6 Fig, and S1 Table):

We addressed this concern in Lines 78-84. We also added a more detailed methodology regarding the RNA pulldown and mass spectrometry experiment in Lines 396-426. The exact sequence of the 205 bp transactivation element was also provided in the S6 Fig. Lastly, we attached the full mass spectrometry result in S1 Table.

**4) What is the justification for using female larvae for cell lysates (line 254) when in lines 51-53 it is stated that DAPALR and Dsx1 alpha are exclusively expressed in males? Even though Shep is expressed in both males and females how could you be sure you are not missing a male specific factor that binds to DAPALR and Dsx1?**

Response:

We agree that we cannot exclude the possibility that there may be a male-specific RNA binding factor that associates with *DAPALR* and *Dsx1*. However, from our previous study, we could not find any RNA binding protein that clearly shows differential expression between males and females in *Daphnia magna* (Nong *et al*, 2020). Thus, we did not consider the sexual dimorphism of the RNA binding protein expression.

**5) No direct evidence is given for the binding of shep to the TGE like element. Can shep pulldown a DAPALR and Dsx1 RNA with the TGE and when the TGE is mutated is this interaction inhibited? Can shep interact with a TGE RNA in vitro as determined by a technique like EMSA?**

Response (Fig 6A and 6B, Lines 264-307):

Thank you for the suggestion. We conducted the *in vitro* translation and luciferase assay to show that Shep only represses *Dsx1* translation in the presence of the TGE-like element. Deletion of the TGE element resulted in no change in the translation efficiency. We also conducted the FLAG pulldown experiment to show that the Shep directly binds to the TGE-like element *in vitro*. In this experiment, we proved that Shep can pull down the RNA with the TGE element and this interaction was inhibited in the absence of the TGE element. These results were shown in Fig 6A and 6B and discussed in Lines 264-307.

**6) Additionally, overexpression of the TGE does not directly support the conclusion that the “TGE like motif is essential for Shep and for DAPALR’s function in regulating Dsx1.” How do you know that the TGE is not sequestering another RNA binding protein required for regulating Dsx1 translation?**

Response (Lines 230-232, Fig 5C, Fig 6A, and 6B):

We agree that we cannot make a conclusion based only on the results of the overexpression of the TGE, hence we revised and toned down the statement in Lines 230-232. To provide more evidence for this claim, we performed additional *in vitro* assays. The suppression experiment using the GFP and luciferase mRNAs *in vivo* and *in vitro* clearly showed that Shep suppresses the *Dsx1* translation only in the presence of the TGE element (Fig 5C and Fig 6A). The translation of the *Dsx1* reporter mRNA with the deleted Shep binding site was not affected by the addition of Shep. The pulldown assay demonstrated binding of the Shep to the TGE (Fig 6B). Taken together, we suggest that the TGE sequesters Shep responsible for repression of the *Dsx1* expression.

**7) As there is no direct evidence presented for shep binding to a TGE the authors can not call the TGE a “shep binding site”. The experiments presented in Figure 5C and 5D are therefore over interpreted when it is stated that “Shep suppresses the translation by binding to the Dsx1α 5´ UTR”. Can you show that shep does not bind or has reduced binding to an RNA without a TGE?**

Response (Line 259, Lines 264-307, Fig 6A, and 6B):

We agree that the *in vivo* results shown in Fig 5C and 5D are not enough to make our claim due to the possibility that other endogenous factors may have contributed to the repression we observed after injection. We have edited this statement in Line 259 accordingly. However, the FLAG pulldown showed that Shep exclusively binds to the RNA harboring the TGE and not in the RNA without the TGE (Fig 6B). Luciferase assay also showed that Shep exclusively represses the translation of the *Dsx1* mRNA with the TGE, while the translation of the *Dsx1* mRNA without TGE remained unaffected (Fig 6A, Line 264-307).

**8) The in vitro luciferase assays presented in Figure 5E require a negative control mRNA in place of the shep mRNA. How do you know that having two mRNAs in the reaction (reporter mRNA and shep mRNA) does not compete for the available ribosomes in the reticulocyte lysate and that is why you have reduced luciferase activity from the reporter mRNA?**

Response (Fig 6C):

Thank you for the suggestion. We added the GFP mRNA that does not contain any TGE-like element in its sequence as a negative control for the *in vitro* luciferase assay which is now in Fig 6C. This proves that the reduced translation observed after the addition of Shep is due to its repression activity and not because of the competition for available ribosomes in the reticulocyte lysate. Moreover, the addition of Shep did not affect the translation of the reporter mRNA that does not have the TGE element. These results showed that having two mRNAs in the reaction did not cause any reduction in the translation efficiency.

**9) Mapes et al 2010 showed that the shep C. elegans ortholog, SUP-26, associates with poly(A)-binding protein 1 (PAB-1) in vivo and may repress tra-2 expression by inhibiting the translation-stimulating activity of PAB-1. Does shep interact with the Daphnia PAB-1?**

Response:

In *Daphnia*, while the TGE element in *Dsx1* was found in the 5´ UTR, it is possible that the 5´ UTR forms a loop with the 3´ UTR and interacts with proteins binding in this region. However, because in our experiment, we only used the *Dsx1* 5´ UTR for the RNA pulldown assay, we did not detect nor we cannot say anything about the binding proteins interacting in the 3´ UTR. This is out of the scope of our current study but this is interesting to investigate in the future.

**10) A CLIP type technique should be used to determine the range of RNAs that shep binds to and determine whether the shep interacting RNAs are mostly mRNAs or are there other ncRNAs that shep can bind to. It would be interesting to see if all these RNAs contained a TGE. This type of analysis would greatly improve the impact of this manuscript. Is DAPALR the sole example of this mechanism of translational regulation or does this regulation occur more commonly?**

Response:

Thank you for the suggestion. We plan to conduct the CLIP experiment in the next step and future direction of our research to show the extent of the Shep function.

Moreover, to address the other concern, we added the discussion in Lines 374-382. A previous study enumerating a list of post-transcription regulators that Shep binds to has been published in *Drosophila* (Olesnicky *et al*, 2018). In this paper, 5 out of 77 Shep targets are noncoding RNAs, none of which mechanisms have been studied extensively. Hence the three-way network of translation regulation between an mRNA, noncoding RNA and binding protein may be the first to be recorded involving Shep and *DAPALR*. But this regulation may occur more commonly since sense-overlapping lncRNAs like *DAPALR* comprise the majority of the present lncRNA (Kuo *et al*, 2017) in addition to broad expression of Shep in different tissue types (Olesnicky *et al*, 2018; Chen *et al*, 2014).

Minor comments

**The manuscript requires improved English language throughout.**

Response:

We apologize for the flaws. We carefully re-checked and edited our manuscript.

**Line 27 – missing word? - determining gene “in” Daphnia magna**

Response (Line 27):

Thank you for pointing this out. We edited it accordingly.

**Lines 32-34 – sentence is confusing/not clear - “These results suggest that the presence of Shep suppresses the unintentional translation of Dsx1 by setting a threshold and expression of the sense lncRNA, DAPALR cancels the suppression.”**

Response (Lines 31-33):

We apologize for the confusion. We revised the statement in Lines 28-31.

**Line 51 – the statement “DAPALR is transcribed from upstream of the transcription start site..” is not clear. Please state what transcription start site you are referring to. I assume it is the Dsx1 alpha transcription start site?**

Response (lines 61-66, Fig 1A):

We apologize for the unclear sentence. Yes, we meant the *Dsx1* alpha transcription start site and we have revised and explained it more in detail in Lines 61-66. We also added Fig 1A to clearly show the genomic organization of *Dsx1* and *DAPALR*.

**Lines 56-57 – the statement “A series of results showed that Shep functions as a suppressor by binding Dsx1 mRNA 57 and DAPALR sequesters the Shep to activate the dsx1 translation.” is vague. Please specifically state what the “series of results” are.**

Response (Lines 67-70):

We revised accordingly and enumerated the “series of results” in Lines 67-70.

**Lines 106-107 – How can you say DAPALR overexpression causes increased Dsx1 mRNA when you have not specifically measured Dsx1 mRNA by RT-qPCR and have only observed increased mCherry signal in Extended Data Figure 3?**

Response:

The *Dsx1* mRNA was measured by RT-qPCR after *DAPALR* overexpression in the previous study (Kato *et al*, 2018). However, this discussion was removed in the current revised manuscript.

**Lines 113-114 – The statement “We used two types of gRNAs targeting each RRM on the Dsx1 reporter strain and obtained a line that has frameshift mutation by 15 nt insertion just before the RRM1 domain” is confusing. This is not a “frameshift” mutation as the correct reading frame is still maintained, it is just an insertion mutation.**

Response (Lines 155-157):

Thank you for pointing this out. We edited it accordingly in Lines 155-157.

**Reference needed for the information given in lines 149-151 – Mapes et al 2010**

Response (Line 217):

Thank you for pointing this out. We added it accordingly.

**Line 181 – this section and Figure 5E describes an in vitro assay so it is confusing when the authors state that “the Shep suppresses the translation of the injected mRNA (Fig. 5e).” This statement needs to be explained better as I believe the authors are referring back to a previous result.**

Response (Lines 269-273):

Thank you for pointing this out. This result is now in Fig 6A and we revised the statement in Lines 269-273.

**Line 201 – “is caused by” should be replaced with “causing”**

Response (Line 321):

Thank you for pointing this out. We edited it accordingly.

**Lines 308-309, line 324 – what were the specific primers used for RT-qPCR of shep, dsx1 and mCherry? Do the dsx1 primers detect all the isoforms?**

Response (S3 Table):

All the primer pairs used for RT-qPCR were enumerated in S3 Table.

**The Materials and Methods section needs to include the detailed methods of how relative fluorescent intensity differences were obtained and calculated for Figures 2B, 4B and 5D.**

Response (Lines 483-492):

We added a more detailed method on how the relative fluorescent intensity differences were measured and calculated in Lines 480-489.

**Figure 5, panels 5D and E – what do the letters “a” “b” “c” represent above each of the bars in the graphs? This information should be provided in the Figure 5 legend.**

Response (Fig 5 legend):

The letters were used to represent the statistical significance of the data. However, we edited the figures to present the statistical significance of the values clearer and we also improved the legends of the figures to address this concern.

**Please define somewhere in the methods what the exact sequences of the DAPALR full and DAPALR partial that are used in Figure 5E.**

Response (S6 Fig):

The sequences of the *DAPALR* full and partial regions used in Fig 6C are presented in S6 Fig.

**Many of the references are missing their Journal, Volume and/or Pages.**

Response:

Thank you for pointing this out. We edited them accordingly.

**Reviewer #2**: In the manuscript Perez et al., the authors identify Shep as an RNA-binding protein of the Daphnia lncRNA DAPALR. Previous work has shown that expression of DAPALR induces the expression of Dsx1, a gene with which it overlaps on the same strand. Both DAPALR and Dsx1 are expressed only in males and are required for male determination. Shep on the other hand is expressed in both males and females. The authors perform a series of experiments using reporter constructs showing that manipulation of Shep levels affect Dsx1 expression but not mRNA levels and conclude that Shep must repress translation of Dsx1 similarly to what has been suggested for the C. elegans Shep homolog Sup-26 translational repression of tra-2. The authors identify a TGE sequence in Dsx1 and DAPALR similar to that identified in tra-2 and perform a series of experiments to suggest that the TGE sequence alone is necessary and sufficient for these effects. Aside from reporter constructs, one in vitro translation experiment is performed in reticulocyte lysate to show that translation of Dsx1 5’ UTR fused to a reporter is sensitive to the presence of Shep and additional DAPALR ncRNA. Overall the results are consistent with the possibility that Shep contributes to translational repression of Dsx1, but no direct biochemical evidence is provided, such as interaction of Shep with translational machinery or changes in Dsx1 mRNA association with translating ribosomes. The results are also consistent with the possibility that DAPALR acts a decoy. The results may be of interest to those studying lncRNA or translation.

Response:

Thank you for reviewing this manuscript. We really appreciate all your suggestions and herewith is our humble and careful response to your comments below.

Specific points:

**1. The authors claim that Shep knockdown results in a pattern that resembles overexpression of DAPALR in females. Do the authors mean males, which is what is shown in Extended Fig. 3?**

Response (Lines 121-122):

The results of the *Shep* knockdown showed the same pattern as of the overexpression of *DAPALR* in both males and females. The *DAPALR* overexpression in female was compared from the previous study (Kato *et al*, 2018), while the male was shown in S3 Fig. We edited the statement in Lines 121-122 to make this clearer.

**2. Why is 2x greater Dsx1 transcript observed in females after Shep knockdown in Fig. 2? The explanation of the yolk doesn’t make any sense to me. Are the authors suggesting that Shep regulates Dsx1 expression through a different mechanism specifically in female yolk?**

Response:

This discussion was removed in the current manuscript. The previous assumption about the *Dsx1* mechanism in the yolk was only inferred because of the intense mCherry signals observed from this region. While this is an interesting idea, we admit that we cannot make any claim regarding this because of lack of evidence and it is out of our scope. Maybe this could be investigated further in the future.

**3. Lack of changes of Dsx1 mRNA expression is used throughout the manuscript to argue that the observed effects on reporter expression are due to changes in translation efficiency. The authors should use a second reference transcript other than ribosomal protein L32 to ensure that their results are correctly calibrated.**

Response (Fig 2C, 2D, 3D, 4C and 4D):

Thank you for the suggestion. For more accurate analysis as recommended, we performed additional qPCR experiments to measure multiple reference gene expression in the same samples of *Shep* knockdown, mutant and overexpression. The *Dsx1* and *Shep* gene expression levels were normalized by the geometric mean of the reference genes (Vandesompele et al. 2002), namely *L32*, *L8*, and *Cyclophilin* as mentioned in the method section in Lines 506-510. The gene expression graphs in Fig 2C, 2D, 3D, 4C and 4D were changed according to our latest analysis.

**4. Why do the authors think that the Shep mutant that they generated in Fig. 3 does not increase Dsx1 mRNA levels in females in the same fashion as the Shep knockdown? Is this related to the differences in developmental stage observed? Why aren’t embryos examined for these mutants as in other figures?**

Response (Lines 172-175):

Unlike in *Shep* knockdown, the female mutant did not exhibit a significant increase in *Dsx1* mRNA levels. This could be due to the fact that the mutation that was induced did not cause any frameshift mutation; only 15 bp was inserted (Fig 3A). The Shep was only impaired of its full function, hence not significantly changing the *Dsx*1 expression level. Complete knockout of the *Shep’s* RRM domain as shown in S5 Fig resulted in abnormal phenotype and they were not able to develop into adults.

Since the established mutation was weak, there were no noticeable differences between the mutant embryos and the wildtype. Hence, adult mutants were used for the observation of the mCherry fluorescence signals. The distinction of the sexually dimorphic parts where *Dsx1* is usually highly expressed is also more evident in the fully matured stage of the *Daphnia*. This was mentioned in Lines 172-175.

**5. In Fig. 5, it would be better to use a scrambled sequence for the TGE rather than a full deletion. “Mutated” is a misleading term.**

Response:

Following your comment, we changed the term “mutated” to “deleted” in the manuscript.

**6. In Fig. 5E, the authors should do controls of addition of DAPALR full or partial in the absence of Shep mRNA to ensure that these are Shep-dependent effects.**

Response (Fig 6C):

Thank you for the suggestion. We added the *DAPALR* full and partial RNAs without the *Shep* mRNA to show that the repression is due to Shep alone. The result is in Fig 6C.

**7. The authors should also consider doing more quantitative experiments to test the decoy model.**

Response (Fig 6D, Lines 309-320):

Thank you for the suggestion. We conducted additional experiments to test the decoy model by performing the suppression and rescue assay using different concentrations of the full region of *DAPALR* and its core element. Results showed that the effect of the full region and core element of *DAPALR* is the same and their efficiency to relieve the repression caused by Shep is dose-dependent. Both the full *DAPALR* and its core element are needed to be added at a concentration at least 5 times higher than the reporter mRNA and Shep to observe its decoy activity. The new result is shown in Fig 6D and discussed in Lines 309-320.

**Reviewer #3:** In the previous study, the authors' group identified a lncRNA called DAPALR overlapping with the 5'-UTR of the Doublesex1 (Dsx1), which is the male determining gene in Daphnia magna. In the present study, the authors identified the Shep protein as a DAPLAR binding protein and provided a several lines of evidence that Shep functions as a supressor by binding Dsx1 mRNA and DAPLAR sequesters the Shep to activate the dsx1 translation.
Overall, this is a nicely done, and several interesting observations are represented and discussed. However, it remains obscure that the importance of the Shep-DAPALR interaction for governing sexual dimorphic expression of Dsx1. This is because the authors did not mention any about whether functional depletion of Shep by RNAi or CRISPR affects sexual dimorphic trait in the text. Such data was given as supplementary figures but I think these figures should move to the main body of the manuscript and the authors should give some explanations and discussions about the phenotype observed in individuals treated with Shep RNAi and individuals with Shep mutations. Thus, I think major revisions as described below are required prior to the publication.

Response:

Thank you for reviewing this manuscript. We really appreciate all your suggestions and herewith is our humble and careful response to your comments below.

Major comments for revision

**1. Fig. 2A. Did the siRNA mediated knockdown of Shep expression affect sexual development? The author should give some explanations about the phenotype observed in the siShep animals by referring extended figure 3B.**

Response (Lines 126-128, Fig 2):

As the first antenna did not show increased mCherry fluorescent signals recapitulating Dsx1 expression in females, we can safely say that no sex reversal was observed as we mentioned in Lines 126-128.

**2. Fig. 2C. The expression level of Dsx1 in the siShep females was more than twice as high as that in control females. Was the level comparable to that in normal males? If so, did the siShep females exhibit partial or complete female-to-male sex reversal? I think the authors should describe about such information to help readers to understand the importance of Shep in sexual differentiation. If the siShep females did not show such sex reversal phenotype, then the authors should give some discussions about why knockdwon of Shep expression did not affect sexual development.**

Response (Lines 126-128, 326-352):

The expression level of *Dsx1* after knockdown in siShep female samples is still not as high compared to the *Dsx1* expression in normal males; hence, no sex reversal could be observed. Because of the ubiquitous mCherry enhancement we observed after *Shep* knockdown in both male and female, we propose the noise-canceling mechanism as the function of Shep and *DAPALR* in sexual differentiation. In this scenario, in females and non-sexually dimorphic tissues, dsx1 would be silenced at the transcriptional level. Shep may represse *Dsx1* at a certain threshold to cancel its unexpected expression and accomplish sexual dimorphic expression. We mentioned this result in Lines 126-128 and discussed this idea in Lines 326-352.

**3. Fig. 2B~2E. The authors described that RT-qPCR results were normalized with L32 expression levels. However, the values shown in the graph are obviously indicated as relative to the gene expression level in siControl animals. The authors should explain this discrepancy.**

Response (Lines 506-511):

The RT-qPCR results were all normalized with the expression levels of the housekeeping genes *L32*, *L8* and *Cylophilin*. The normalized gene expression level of the treated samples are then relatively compared to the normalized expression level of the control. We edited the methods to address this in Lines 506-511.

**4. Lines 106-109. Shep RNAi and DAPALR overexpression in embryos caused increased level in Dsx1 expression in yolk cells. The authors proposed that this may be due to the unique system specialized for energy metabolism in yolk cells. But I don't think this explanation is reasonable for the increased level of Dsx1 expression in yolk cells. The authors should propose more detailed and reasonable mechanisms for how Dsx1 expression was increased by Shep RNAi and DAPALR overexpression in yolk cells.**

Response:

This discussion was removed in the current manuscript. The previous assumption about the *Dsx1* mechanism in the yolk was only inferred because of the intense mCherry signals observed from this region. While this is an interesting idea, we admit that we cannot make any claim regarding this because of lack of evidence and it is out of our scope. Maybe this could be investigated further in the future.

**5. Lines 113-114. In the present study, several lines with mutations in Shep gene were establiShep by CRISPR/Cas9 system. How did the authors rule out the possibility of off-targeting effect. They should provide evidence to prove that the phenotype observed in the Shep mutants were not due to the off-targeting effect.**

Response (Lines 438-446):

Only one mutant line was generated in the experiment (Fig 3A, 2B, 2C). The gRNAs targeting the two RRMs were carefully designed and their specificity to *Shep* could avoid off-target effects because the DNA region with up to five base pair mismatches with the gRNA is susceptible to editing by the Cas9/gRNA complex (Fu *et al*, 2013; Jiang *et al*, 2013). This was mentioned in the methods in Lines 438-446.

**6. Fig. 3C. The Shep mutation caused increased level of Dsx1 expression in females. Did the Shep mutant females show partial or complete female-to-male sex reversal? The author should clarify this point by referring Extended Data Figure 4 that shows phenotype of the Shep mutants. If the the Shep mutant females did not exhibit any abnormalities in sexual development, then the authors should give some reasons.**

Response (Lines 188-192):

The Shep mutant displayed mCherry signals in its whole body especially the appendages. High expression of mCherry signal could also be observed in the first antennae that is one of the major male-specific traits. However, as shown in Fig 3C, one of the major male traits, first antennae, did not develop elongated like in males. In addition, shape of the carapace was not masculinized, signifying that sex reversal and male differentiation did not occur. S5 Fig (formerly Extended data Figure 4) is not suitable for analyzing male-specific development because embryos injected with Cas9 and Shep-targeting gRNAs exhibited deformity during as reported in *Drosophila* and other species (Lines 188-192).

**7. Fig. 3C, dotted lines. The authors said that the red signal from the guts surrounded by the dotted lines represents the autofluorescence of the food used in daphnia rearing. Then, similar red signal should be observed in males. Why was the red signal only observed in females?**

Response:

The feeding activity and time of taking the photos were different between the female and male samples hence the difference in the red signal caused by the autofluorescence of the chlorella could be observed.

**8. Fig. 3D. As shown in Fig. 2C, Shep knockdown caused increased level of Dsx1 expression in female embryos. On the other hand, Shep mutation did not cause such increment in Dsx1 expression level in females. The authors should explain this discrepancy.**

Response (Lines 172-175):

Unlike in *Shep* knockdown, the female mutant did not exhibit a significant increase in *Dsx1* mRNA levels. This could be due to the fact that the mutation that was induced did not cause any frameshift mutation; only 15 bp was inserted (Fig 3A). The Shep was only impaired of its full function, hence not significantly changing the *Dsx1* expression level. Complete knockout of the Shep’s RRM domain as shown in S5 Fig resulted in abnormal phenotype and they were not able to develop into adults.

Since the established mutation was weak, there were no noticeable differences between the mutant embryos and the wildtype (S4 Fig). Hence, adult mutants were used for the observation of the mCherry fluorescence signals. The distinction of the sexually dimorphic parts where *Dsx1* is usually highly expressed is also more evident in the fully matured stage of the *Daphnia*. This was mentioned in Lines 172-175.

**9. Lines 128-129. The authors conclude that Shep controls Dsx1 expression at post-transcriptional levels. However, their previous study reported that Dsx1 mRNA level in females was much lower than that in males. If so, the protein level of Dsx1 should be still lower in females than in males even though post-transcriptional suppression by Shep is disrupted by the Shep mutation. The authors should give some explanations about this discrepancy.**

Response (Lines 326-352):

Yes, it is true that with or without the post-transcriptional suppression caused by Shep, the protein level of Dsx1 remains lower in females than in males because the *Dsx1* mRNA level of females is already lower than in males, to begin with. While Shep does not function to bring the *Dsx1* expression to a degree high or low enough to cause sex reversal, we propose that the function of Shep is to limit the *Dsx1* expression at a certain threshold to cancel noisy and unexpected expression to aid in sexual dimorphism or male differentiation. We discussed this proposed mechanism in Lines 326-352.

**10. Extended data Figure 4A. The sex of each individual in the photo should be clearly stated. Also, the authors should make clear whether these individuals show abnormalities in the sexual dimorphic traits. Was the sexual difference in the expression pattern of Dsx1 in these mutants disrupted? The authors should also give some explanations about this to verify whether Shep is indeed responsible for sexual dimorphic expression of Dsx1 protein.**

Response (S5 Fig):

All the individuals in the photos are female. The Cas9 protein and target gRNAs were all injected into female eggs. The sexual dimorphic traits could not be observed among the samples in this S5 Fig because they did not develop further except for the group with “normal development”. They were cultured to screen for germline transmission of the mutation and establishment of the mutant line. The rest of the daphniids with other phenotypes (delayed development, deformed embryos and unhatched eggs) exhibited somatic mutations and died prematurely before the sexual difference could be evident.

**11. Extended data Figure 4B. The images described in this figure are uncomfortable. The authors should load the PCR products on the same gel to precisely compare the size of each band and show a full-picture of the gel to show that each band representing deletion or insertion mutation was specifically observed in the mutant animals. Also the position of the asterisk should be modified to exactly next to the band (in the present version, several asterisks are positioned at where there is no band). The authors should also show the nucleotide sequences of the deletion and insertion mutations as shown in Fig. 3A and clarify whether these mutations cause a frame-shift mutation or not.**

Response:

As recommended, we loaded the PCR products on the same gel for each gene target and the new pictures of the gels are shown in S5B Fig. The extracted daphniids in each sample were pooled based on their displayed phenotype (normal development, delayed development, deformed embryos and unhatched/dead eggs). In this experiment, only embryos injected with Cas9 RNA were used, meaning that G0 individuals were used for genotyping. Thus, the samples from this experiment only showed somatic mutation. Different mutations could be induced in different cells depending on the timing of the Cas9 cleavage; hence, the presence of multiple bands and because of this we could not check the exact sequence of the deletion and insertion mutations.

Minor comments for revision

**1. The entire text and all figures. Regarding the notation of "Shep", the gene symbol should be written as "Shep" in italic notation. The "Shep" notation is only acceptable when it means Shep protein. The same applies to Dsx1.**

Response:

Thank you for pointing this out. We carefully re-checked our manuscript regarding the gene names.

**2. Fig. 2A, 3C, 4A, 5B, and Extended data Figure 5A and 5B. Adding a bright field image will help the reader understand the results.**

Response:

Thank you for the suggestion. We added the bright field images for Fig 2A, 3C, 4A, 5B, S3 Fig and S4 Fig.

**3. Fig. 3A. Not only show the amino acid sequence encoded by the insertion mutation, but also show the amino acid sequence encoded by the wild-type Shep gene.**

Response:

Thank you for the suggestion. We added the amino acid sequence of the wild type *Shep* gene in Fig 3A.

**4. Line 153. Remove "we overexpressed" because it's redundant in the sentence.**

Response:

Thank you for pointing this out. We edited it accordingly.
