# Supplementary material for: Sense-overlapping lncRNA as a decoy of translational repressor protein for dimorphic gene expression
Source: PLoS Genet. 2021 Jul 28;17(7):e1009683. doi: 10.1371/journal.pgen.1009683 (PMC8351930; doi:10.1371/journal.pgen.1009683)
Supplement: Attachment — Submitted filename: Response to reviewers.docx [file pgen.1009683.s011.docx]

**Responses to Reviewers**

**Reviewer #1:** **The authors have provided an improved manuscript and have addressed almost all my concerns sufficiently with revised text and additional experiments and controls. However, a "direct" interaction between shep and the DAPALR or Dsx1 RNA has still not been proven. The new in vitro pulldown assays (Figure 6B), used to prove a direct interaction, contain rabbit reticulocyte lysate so there is still the possibility that the interaction between shep and the TGE containing RNA is not direct. As such, the authors still cannot convincingly say that there is not another protein from the reticulocyte lysate that may be bridging the interaction between shep and the RNA. There are two experiments the authors could include that would prove a direct interaction between shep and a TGE containing RNA. The first experiment, which was suggested in my previous review, would be to take only purified shep and only a TGE containing RNA and see if they interact in vitro using a technique like EMSA. Alternatively, the authors can repeat the pull down assays they have included here in Figure 6B but include a UV crosslinking step (to capture any direct RNA-protein interactions) and then pull down under conditions that disrupt any indirect interactions (ie with detergent and high salt).**

**Response:**

Thank you for all your insights and suggestions. We performed the RNA pulldown with the additional UV crosslinking step and used a High-salt wash buffer [50 mM Tris-HCl (pH 7.4), 1 M NaCl, 1 mM EDTA, 1% Igepal CA-630, 0.1% SDS, 0.5% sodium deoxycholate] to disrupt any unspecific binding. To test the effectivity of the wash buffer and to determine the optimal time for UV exposure, we performed the preliminary experiment using the Shep-FLAG and RNA with TGE at different UV exposure times. As shown below, there was highest enrichment at 90s irradiation; hence we used this for the actual experiment. The results below also showed the effectivity of the washing as it removed the binding without (0s) and at insufficient time (30s) of crosslinking. Results from the final experiment replaced Fig 6B and the methods were modified in Lines 584-590.

| Exposure time to UV (sec) | Normalized expression level |
| --- | --- |
| 0s | 12.5273 |
| 30s | 98.7234 |
| 90s | 1739.18 |
| 270s | 523.26 |
| 810s | 135.231 |

**Reviewer #2: The authors addressed the majority of my previous concerns, but the authors still do not show evidence that Shep directly binds endogenous Dsxl transcripts. The new in vitro binding results do support their hypothesis.**

**Response:**

Thank you for your comments and suggestions. To provide more evidence that Shep directly binds to *Dsx1*, we performed the RNA pulldown once again and revised the protocol to add a UV crosslinking step, and used a stringent High-salt wash buffer to disrupt any unspecific interactions. The methods are revised in Lines 584 to 590 and the new result is shown in Fig 6B.

Thank you once again. Herewith is our humble and careful response to your other comments below.

**I found that their responses to several of Reviewer #3’s comments were not adequately addressed:**

**A)
5. Lines 113-114. In the present study, several lines with mutations in Shep gene were establiShep by CRISPR/Cas9 system. How did the authors rule out the possibility of offtargeting effect. They should provide evidence to prove that the phenotype observed in the Shep mutants were not due to the off-targeting effect.**

**Response (Lines 438-446):** Only one mutant line was generated in the experiment (Fig 3A, 2B, 2C). The gRNAs targeting the two RRMs were carefully designed and their specificity to Shep could avoid off-target effects because the DNA region with up to five base pair mismatches with the gRNA is susceptible to editing by the Cas9/gRNA complex (Fu et al, 2013; Jiang et al, 2013). This was mentioned in the methods in Lines 438-446.

**More than one mutant line should be examined to ensure that off target effects are not an issue.**

**Response:**

Thank you for your comment and suggestion. We did attempt to generate more than one mutant line; but, as we mentioned in Lines 189-193, the other mutants died and failed to develop normally. However, although one line can only be established, it can be observed from S5 Fig that the delayed and deformed samples with somatic mutations also showed the ubiquitous mCherry signals in their whole body same as the established Shep mutant line and Shep-silenced embryos. Although we could not check the off-target effect of the CRISPR/Cas9 among these samples, the bright ubiquitous mCherry fluorescence observed in their whole body can be correlated to the mutations in their Shep RRM domain.

**B)
7. Fig. 3C, dotted lines. The authors said that the red signal from the guts surrounded by the dotted lines represents the autofluorescence of the food used in daphnia rearing. Then, similar red signal should be observed in males. Why was the red signal only observed in females?**

Response: The feeding activity and time of taking the photos were different between the female and male samples hence the difference in the red signal caused by the autofluorescence of the chlorella could be observed.

**Compared photos should be taken with the same exposures. Alternatively, the relative exposure times could be indicated.**

**Response:**

Thank you for your comment and suggestion. We did take the photos under the same exposure conditions. We added this information in Lines 485-489.

**Additional minor comments:**

**C) Authors claimed multiple times that Dsxl transcripts are exclusively expressed in males but in fact there are transcripts and translated RFP in females.**

**Response:**

Thank you for your comment. We revised the claim in Line 64 by changing the word “exclusively” to “highly” as there is still *Dsx1* expression in females even at low levels.

**D) There is no evidence Shep expression peaks at 30hpo, line 111 and there is a mis-callout of figure 1D, line 110.**

**Response:**

Thank you for pointing this out. We revised the citation in Line 111. We also revised the statement about the *Shep* expression increasing after 30hpo in Lines 111-112.

**E) The Shep mutant has additional 5 amino acids, which lead to a 1% increase of the protein size. In figure 3B, it is way more than 1%.**

**Response:**

The bands in Figure 3B are from PAGE analysis of the PCR products after genotyping the Cas9/gRNA targeting region in the Shep coding sequence of the wildtype and the Shep mutant line. The mutant sample showed 15 nt insertion from the wildtype sequence.

**F) Do any Shep antibodies exist that can be used to validate the manipulation of Shep expression?**

**Response:**

Unfortunately, there are no Shep antibodies available to be used in the experiments.

**G) The authors need better discussion of their decoy model – if the lncRNA is expressed at only 10% of Dsxl transcripts and sufficient to function as a decoy, how would Shep be able to inhibit all remaining Dsxl?**

**Response:**

We are thinking that the region of DAPALR expression may be more limited than that of the Dsx1 expression. In this case, it may be reasonable to detect DAPALR expression at only 10% of Dsx1 transcript levels because we extracted total RNA from whole embryos.

In our scenario, DAPALR may be expressed in cells only at the early stage of male differentiation and decrease the threshold of Dsx1 expression by sequestering Shep protein. Then, the translated Dsx1 protein may activate own promoter by positive feedback loop and Dsx1 expression can be maintained. Because *Shep* seems to be ubiquitously expressed in different tissues in the whole body, this RBP would be able to silence Dsx1 expression in the non-sexually dimorphic tissues that do not express DAPALR. Consistent with this hypothesis, when we overexpressed DAPALR ubiquitously or Shep expression was silenced by siRNA, dsx1 reporter mCherry expression was observed throughout the body even in the non-sexually dimorphic tissues.

Because we still don’t have enough experimental evidence to prove this scenario, we did not include it in the discussion but this is important for our future study.

**H) If the lncRNA indeed works as a decoy, does its expression fluctuate with Shep and/or Dsxl expression in order to properly decoy?**

**Response:**

We understand that knowing the stoichiometry of the expression of *DAPALR*, *Shep* and *Dsx* would allow us to understand the decoy mechanism better. However, we do not have enough evidence to show this as of the moment but we will investigate this in the future.

**I) Authors should explain briefly why they chose Shep and CUGBP1 while there are a lot of other factors pulled downed as enriched.**

**Response:**

Thank you for the comment. We chose Shep and CGUBP1 among the other proteins that were pulled down because only the two proteins showed high significant p-values. The other proteins that were pulled down also bound with the negative bait RNA used. We mentioned this in Lines 82-85.

**J) In figure 4A, why isn’t the RFP expression symmetrical?**

**Response:**

The asymmetry in the photos taken is because of the difference in angle and direction of the embryos while taking photos. We apologize for this is inevitable because of the difficulty in fixing a living embryo that will still be used in further tests.

**K) What are the RNAs used on line 300? Are they Dsxl-related?**

**Response:**

The RNAs used in the pulldown experiment in Line 300 were the Luciferase reporter mRNA with and without the TGE element. We revised this in Line 301 and in the method section in Line 582.

**L) Why do the authors use images from different angles/stages for almost all figures?**

**Response:**

As I mentioned above, it is rather difficult to take photos of living embryos from the same angle and direction. About the difference in stages, we used embryos at different stages according to the respective experiment. In Fig 2A, we showed the embryos taken at 48hrs because the siRNA used for the knockdown experiment is stable and the effect of Dsx1 and mCherry expression is more evident at later stages. On the other hand, in Fig 3C, we showed the adult Shep mutants because there were no noticeable differences between the mutant embryos and the wildtype (S4 Fig) due to the weak mutation. Also, the distinction of the sexually dimorphic parts where *Dsx1* is usually highly expressed is more evident in the fully matured stage of the *Daphnia*. This was mentioned in Lines 173-175. Finally, in Fig 4A, Fig 5B, and Fig 5C, we used the emrbyos at 30hpo since mRNAs degrade faster and are not as stable as siRNAs so we chose to observe these experiments at an earlier stage.

**M) Figure 5 still shows the term “mutated”.**

**Response:**

Thank you very much. We have changed the term “mutated” to “deleted” in Figure 5.

**Reviewer #3:** I think the revised manuscript is now suitable for publication. I am satisfied with the author's responses and plausible explanations.

**Editor's Comments:**

**Experimental proof of the direct interaction between Shep and Dsx1 RNA should be included in the revised version.**

**Response:**

Thank you for the comment. As suggested by Reviewer #1, we performed the RNA pulldown with the additional UV crosslinking step and used a High-salt wash buffer to disrupt any unspecific binding. The methods were revised in Lines 584 to 590 and the new result was shown in Fig 6B. We hope that this is enough evidence to prove the direct interaction between Shep and *DAPALR* or *Dsx1*.
