# Supplementary material for: Sense-overlapping lncRNA as a decoy of translational repressor protein for dimorphic gene expression
Source: PLoS Genet. 2021 Jul 28;17(7):e1009683. doi: 10.1371/journal.pgen.1009683 (PMC8351930; doi:10.1371/journal.pgen.1009683)
Supplement: Attachment — Submitted filename: Response to reviewers.docx [file pgen.1009683.s012.docx]

**Responses to Reviewers**

**Reviewer #2: The authors have satisfied the majority of reviewer concerns. Minor comments are indicated below:**

**E) The authors need to label ladders.**

**Response:** Thank you very much for carefully reviewing the manuscript and for this suggestion. We labeled the ladder in Fig 3B and reuploaded the new image.

**G) The authors need to include something like their response to reviewers in the Discussion.**

**Response:** Thank you for your comments and suggestion. We added our previous response in the discussion in Lines 350-366.
